# Self-regulation as a stronger predictor than motivation of translation competence: a mixed-methods study of undergraduate translation students

**DOI:** 10.3389/fpsyg.2025.1582455

**Published:** 2025-09-30

**Authors:** Dan Xu, Amily D. Wang Guénier

**Affiliations:** ^1^School of Advanced Translation and Interpretation, Dalian University of Foreign Languages, Dalian, China; ^2^School of Global Affairs, Lancaster University, Lancaster, United Kingdom

**Keywords:** self-regulation, motivation, translation competence, flipped classroom, undergraduate translation students

## Abstract

**Introduction:**

This study moves beyond the traditional focus on motivation to critically examine the dominant role of self-regulation in shaping translation competence within a flipped classroom setting.

**Methods:**

A mixed-methods design was employed with data collected from 131 undergraduate translation students through questionnaires, translation tests, and interviews. Regression analyses were used to assess the predictive strength of motivation and self-regulation, and qualitative interviews provided further insights into students’ learning experiences.

**Results:**

The findings reveal that while motivation initiates learner engagement, it is self-regulation—encompassing goal-setting, self-monitoring, and strategic adaptation—that ultimately determines students’ ability to navigate the complex demands of translation tasks. Regression results confirmed that self-regulation is a stronger and more consistent predictor of translation competence than motivation. Qualitative data further showed that highly motivated students without self-regulatory strategies often struggled to succeed.

**Discussion:**

These results highlight the limitations of motivation alone and call for a paradigm shift in translator education. Pedagogical models should move beyond motivation enhancement to explicitly cultivate learners’ self-regulation skills. Embedding cognitive and metacognitive training into translation curricula can better prepare students for the complexities of professional practice. This study contributes empirical evidence and pedagogical insights to both translation studies and educational psychology, advocating the development of strategic, self-directed translation professionals.

## 1 Introduction

Translation education has increasingly recognized the critical role of cognitive and metacognitive factors in shaping students’ professional competencies. Among the various cognitive and affective factors influencing learning, motivation and self-regulation have been widely recognized as key determinants of academic achievement and skill development (Pintrich and De Groot, 1990; Zimmerman, 2002; Dörnyei and Ushioda, 2021). While multiple cognitive, linguistic, and contextual factors may contribute to the development of translation competence, motivation and self-regulation are two central constructs that have been widely recognized in educational psychology. Motivation provides the necessary drive for students to initiate and sustain effort in learning tasks, while self-regulation equips them with strategies to monitor and refine their learning processes, ensuring long-term success (Pintrich and De Groot, 1990). Motivation determines the effort, persistence, and engagement of students in translation tasks (Dörnyei and Ushioda, 2021), while self-regulation enables them to strategically plan, monitor, and evaluate their learning processes (Zimmerman, 2000). The interplay between these factors is particularly relevant in the field of translation, which demands sustained cognitive effort, problem-solving skills, and adaptive strategies (Hu et al., 2021). Despite their recognized importance, how motivation and self-regulation jointly contribute to the development of translation competence remains an underexplored area in translation education research.

Traditional translation pedagogy has long emphasized linguistic proficiency and technical accuracy, often overlooking the cognitive and affective dimensions of learning (Kiraly, 2014; Beeby et al., 2011; Shreve, 2017). Recognizing this gap, Göpferich and Jääskeläinen, 2009 proposed a comprehensive competence model that places motivation as a key factor underlying translation competence. Robert et al. (2017) subsequently expanded Göpferich’s framework by incorporating additional cognitive, affective, and situational dimensions, further enriching our understanding of translator competence development. While motivation has been widely acknowledged as a key driver of translation learning (Pym et al., 2013; Liu and Yu, 2019; Hurtado Albir and Taylor, 2015), recent studies suggest that motivation alone may not be sufficient to sustain high-level translation performance unless accompanied by effective self-regulatory strategies (Zimmerman and Kitsantas, 2014; Wen et al., 2023; Hadwin et al., 2025; Lu et al., 2022). Similarly, while self-regulation is essential for fostering autonomy and adaptability, it cannot operate in isolation without the motivational drive that initiates and sustains engagement in learning tasks (Eccles and Wigfield, 2020; Pintrich et al., 1991; Zimmerman and Schunk, 2011). Therefore, a comprehensive approach to translation education must integrate both motivation and self-regulation to optimize students’ translation competence.

The flipped classroom model, defined as an instructional approach that reverses the traditional learning sequence by asking students to engage with core material before class while using class time for interactive, problem-solving tasks (Bergmann and Sams, 2012), presents a promising pedagogical framework for fostering motivation and self-regulation simultaneously. By shifting learning responsibilities to students before class and utilizing in-class sessions for interactive applications, the flipped approach encourages self-directed learning while also leveraging students’ intrinsic and extrinsic motivation (Nguyen, 2021). While the flipped classroom model has been increasingly applied in translator training, most existing studies remain focused primarily on linguistic development, translation strategies, and the use of computer-assisted translation (CAT) tools (Beeby et al., 2011; Liu and Yu, 2019; Yang and Wang, 2023; Pietrzak, 2018; Hurtado Albir, 2015; Valero-Garcés and Cedillo Corrochano, 2018; Xiao, 2022). For example, Hurtado Albir (2015) explored competence acquisition processes, Valero-Garcés and Cedillo Corrochano (2018) examined legal translation pedagogy, and Xiao (2022) investigated flipped learning’s impact on translation skills in a Business English context. Despite these valuable contributions, there remains comparatively little attention to psychological processes that support student learning in flipped environments—such as how motivation and self-regulation interact to enhance translation competence. This oversight constrains our understanding of how flipped pedagogy can foster learner autonomy, metacognitive awareness, and sustained engagement in translation tasks. Therefore, this study explicitly addresses this critical gap by focusing on motivation and self-regulation as core psychological constructs within flipped-classroom translator education. Third-year undergraduate students were chosen as the focal population for this study due to their prior exposure to conventional classroom instruction in the first two academic years, which provided a stable baseline for comparison with the flipped classroom model. At this stage, students are expected to demonstrate increasing autonomy in their learning, making them especially relevant for investigating the development of self-regulation and motivation. The flipped classroom approach, by shifting the responsibility of content engagement to pre-class stages, is designed to stimulate self-directed learning behaviors and strengthen both motivational and self-regulatory capacities. The current study specifically explores how these two psychological constructs interact to shape translation performance by adopting a mixed-methods approach to provide empirical evidence on the interplay between motivation, self-regulation, and translation competence. By clarifying the mechanisms through which these factors contribute to translation performance, this research aims to inform the design of more effective pedagogical strategies that incorporate cognitive, metacognitive, and affective dimensions of learning. Ultimately, this study contributes to a more holistic understanding of translator education, advocating for a balanced and student-centered approach that integrates motivation and self-regulation to enhance translation learning outcomes.

## 2 Literature review

### 2.1 Translation competence

Translation competence refers to the ability to carry out translation tasks to a professional standard, involving not only linguistic skills but also cognitive, strategic, and instrumental capacities (Beeby et al., 2011; EMT, 2022). It is widely recognized as a multidimensional construct, incorporating sub-competences such as bilingual proficiency, cultural knowledge, transfer strategies, technological literacy, and self-regulation. Translation competence has been extensively studied in translation studies, with scholars emphasizing its multidimensional nature (Beeby et al., 2011; Hurtado Albir et al., 2022; PACTE Group, 2017; Hurtado Albir et al., 2022). While early models conceptualized translation competence as a subset of linguistic proficiency (Wilss, 1996), more recent frameworks recognize cognitive, strategic, and affective components as integral to professional translation (Kiraly, 2000; EMT, 2009; EMT, 2022). For instance, the EMT competence framework (2022) further highlights digital literacy and technological competence as essential skills for modern translators.

In particular, post-editing has emerged as an important area within translation competence development. Nitzke and Hansen-Schirra (2021) provide a concise guide to post-editing practice and the competencies it requires, reflecting the increasing relevance of this skill set in professional contexts. Likewise, Yang and Wang (2023) examine the interplay of self-regulation, critical thinking, and motivation in predicting students’ performance in machine translation post-editing, emphasizing how cognitive and affective factors are central to contemporary competence models.

The PACTE model (see Beeby et al, 2011) categorizes translation competence into various sub-competencies, including linguistic-cultural competence, translator competence, and translation service provision competence, all of which require strategic decision-making, motivation, and self-monitoring skills, with strategic decision-making positioned as a core sub-competency (PACTE Group, 2017; Beeby et al., 2009). Similarly, Shreve (2017) argues that translation is a complex cognitive task that necessitates reasoning, problem-solving, and metacognitive reflection. Given these cognitive demands, researchers have increasingly called for pedagogical approaches that explicitly train students in both motivational strategies and self-regulatory learning behaviors to enhance translation competence (Hu et al., 2021; Pietrzak, 2021; Valero-Garcés and Cedillo Corrochano, 2018; Xiao, 2022; Yang and Wang, 2023), reflecting a broader trend across contemporary translator education.

### 2.2 Motivation

Motivation generally refers to the psychological factors that initiate, direct, and sustain goal-oriented behaviors, influencing the learners’ persistence and intensity in engaging learning tasks (Deci and Ryan, 2000; Dörnyei and Ushioda, 2021). It is generally categorized into intrinsic motivation, driven by interest and enjoyment, and extrinsic motivation, influenced by external incentives like grades and career goals (Dörnyei and Ushioda, 2021). Intrinsic motivation fosters deeper learning and creativity, whereas extrinsic motivation sustains engagement, especially in structured learning environments (Ryan and Deci, 2000; Deci and Ryan, 2013; Ryan and Deci, 2020; Ryan and Deci, 2023; Ryan and Deci, 2024; Ryan et al., 2021). Motivation is consistently regarded as fundamental to successful learning outcomes; as Pintrich (2003) explicitly states, motivation serves as the cornerstone of effective self-regulated learning, profoundly impacting students’ cognitive, behavioral, and emotional engagement. Widely acknowledged as a crucial determinant of learning engagement, motivation has recently been reconceptualized in more insightful, dynamic terms within educational psychology and translator education. Traditional motivation theories, such as Self-Determination Theory (SDT) proposed by Deci and Ryan (2000), have evolved significantly in recent studies. For instance, Ryan and Deci (2023) emphasized that motivation must be understood contextually as a dynamic interaction of intrinsic (e.g., enjoyment, personal interest) and extrinsic factors (e.g., external rewards, academic achievements), particularly within highly specialized cognitive domains such as translation. Similarly, recent advances in Expectancy-Value Theory (EVT) have highlighted learners’ perceptions of task relevance, interest, and utility value as key predictors of sustained engagement and deep learning (Eccles and Wigfield, 2023).

In translation education, motivation plays a crucial role in sustaining students’ engagement and perseverance in acquiring translation skills (Liu and Yu, 2019). Given the cognitively demanding nature of translation, motivation alone may not be sufficient; it must be supported by self-regulation strategies to optimize learning outcomes (Zimmerman, 2002). When students recognize the long-term benefits of translation training, they are more likely to sustain effort and persistence. Therefore, fostering motivation alongside self-regulation is essential for long-term skill development in translation education. Given the cognitively demanding nature of translation, sustained motivation is essential for developing linguistic, cultural, and technical competencies. However, while motivation is a key driver of learning behaviors, research suggests that it must be complemented by effective self-regulation strategies to optimize learning outcomes (Zimmerman, 2002; Schunk and Zimmerman, 2012a, 2012b; Schunk and Zimmerman, 2023; Zimmerman, 2023; Zimmerman et al., 2023). Furthermore, task value beliefs, which reflect students’ perceptions of the relevance of translation skills to their future careers, have been found to significantly impact motivation levels (Wang and Zhang, 2024). Recent research has emphasized additional psychological constructs that influence student motivation. For instance, del Mar Haro-Soler (2018) emphasized the importance of self-confidence for maintaining motivation in translator training. Haro-Soler and Kiraly (2019) showed that collaborative pedagogical approaches that build students’ self-efficacy beliefs enhance persistence and engagement. Singer and Soler, 2022 provided further evidence that translation practice itself significantly impacts students’ self-efficacy beliefs, thereby reinforcing motivation. In addition, Singer (2022) found that translator identity development plays a crucial role in sustaining learners’ long-term commitment and engagement with translation education. Together, these studies suggest that beyond intrinsic and extrinsic motivation, constructs like self-efficacy, self-confidence, and identity are critical to maintaining persistence and performance in cognitively demanding translation programs.

When students recognize the long-term benefits of translation training, they are more likely to sustain effort and persistence. Students with a mastery orientation, who focus on skill development and understanding, tend to demonstrate higher motivation and deeper learning engagement. In contrast, those with a performance orientation, who prioritize grades and external rewards, may experience fluctuating motivation levels, particularly when facing cognitively demanding translation tasks and challenges (Yang and Wang, 2023).

Research on computer-assisted translation (CAT) tools suggests they can enhance motivation by reducing cognitive load, but may also create over-reliance, affecting intrinsic motivation (Liu et al., 2022). These dynamics illustrate that sustaining motivation in translator training contexts depends not only on external tools but also on learners’ cognitive and metacognitive strategies. While motivation plays a critical role in initiating engagement, sustaining effort over time requires additional cognitive strategies. In particular, learners must develop the ability to regulate their motivation by setting realistic goals, monitoring progress, and adapting strategies when faced with challenges (Schunk and Usher, 2019). This transition from motivation-driven engagement to self-regulated learning is particularly relevant in translation education, where tasks demand prolonged concentration and adaptability. Without self-regulation, even highly motivated learners may struggle with complex translation tasks due to the lack of strategic learning approaches (Zimmerman and Kitsantas, 2014). Thus, the intersection between motivation and self-regulation is crucial for fostering long-term success in translation training. While these tools can enhance motivation by reducing cognitive load and increasing translation efficiency, they may also lead to over-reliance on technology, potentially diminishing intrinsic engagement in translation learning. This highlights the need for balanced pedagogical approaches that integrate motivation-enhancing strategies with cognitive skill development.

### 2.3 Self-regulation

Self-regulation is a multidimensional process that enables learners to manage cognitive, metacognitive, and motivational resources to optimize learning outcomes (Zimmerman, 2002). It involves goal-setting, self-monitoring, and adaptive strategy use, ensuring persistence in challenging tasks (Pintrich, 2004). Self-regulated learners exhibit higher autonomy, problem-solving skills, and academic performance across disciplines (Schunk and Usher, 2019).

Zimmerman (2002) defines self-regulation as a cyclical process encompassing three phases: forethought, performance, and self-reflection. In the forethought phase, learners set goals and select strategies. During the performance phase, they implement strategies while monitoring progress. The self-reflection phase involves evaluating outcomes and refining techniques for future improvement (Panadero, 2017). This iterative process enhances learners’ ability to manage complex tasks effectively.

In translation education, self-regulation is critical due to the cognitive demands of translation tasks, which require ongoing problem-solving, decision-making, and metacognitive reflection (Shreve, 2017). Self-regulated learners actively plan translations, monitor textual coherence, and refine strategies based on feedback, making them more adaptable in handling linguistic complexities (Pietrzak, 2018; Hu et al., 2021). Research indicates that students with strong self-regulation skills demonstrate greater efficiency, higher task persistence, and improved translation competence (Schunk and Zimmerman, 2012a, 2012b; Yang and Wang, 2023). Similarly, recent research by Bolaños-Medina and Núñez (2022) emphasized the pedagogical necessity of explicitly training translation students in SRL strategies. Their findings indicated that learners who actively engaged in strategic goal-setting, systematic self-monitoring, and structured reflection consistently demonstrated superior performance on challenging translation tasks compared to peers relying solely on motivational drive. Thus, contemporary translation pedagogy increasingly emphasizes explicit training in self-regulatory strategies to complement motivational support, reflecting a deeper understanding of the cognitive demands inherent in translation tasks (Hu et al., 2021; ZabihiAtergeleh et al., 2025).

Despite its significance, self-regulation does not develop spontaneously; it requires deliberate instructional support. Pedagogical strategies such as structured goal-setting, reflective translation journals, and scaffolded feedback mechanisms have been shown to enhance students’ability to self-regulate effectively (Panadero, 2017; Yang and Wang, 2023). Embedding self-regulation training into translation curricula fosters greater learner autonomy and equips students with the necessary skills to navigate translation challenges in professional practice. Encouraging metacognitive awareness through self-assessment rubrics and strategic learning interventions can further enhance students’ adaptability and long-term success in translation learning.

### 2.4 The flipped classroom

The flipped classroom refers to an instructional model that inverts the traditional learning structure by delivering instructional content, often online, outside of the classroom, while in-class time is dedicated to interactive, student-centered activities (Abeysekera and Dawson, 2015; Nguyen, 2021). The flipped classroom model represents a shift from passive to active learning, promoting student autonomy and engagement (Abeysekera and Dawson, 2015). By requiring students to engage with learning materials before class, the flipped approach encourages goal-setting, time management, and self-monitoring (Nguyen, 2021). Studies have shown that students in flipped translation classrooms exhibit higher levels of both motivation and self-regulated learning behaviors compared to those in traditional settings (Bagherzadeh et al., 2021; Abeysekera and Dawson, 2015; Nguyen, 2021; Nguyen et al., 2022; Muali et al., 2023; Omarchevska et al., 2024). However, challenges such as cognitive overload and lack of structured self-assessment tools can hinder students from fully benefiting from the flipped model (Colomo-Magaña et al., 2020). Addressing these challenges requires a deliberate integration of motivational and self-regulation strategies into flipped translation curricula, ensuring that students develop the necessary cognitive and affective skills to manage independent learning effectively.

While motivation and self-regulation have been studied independently in translation education, few studies have examined their combined impact on translation competence. Among these few, Ameri and Ghahari (2018) found that intrinsic motivation fosters self-regulatory learning behaviors that contribute indirectly to translation performance, while Bagherzadeh et al. (2021) observed that flipped classroom designs can enhance both motivation and self-regulation among novice translators. Yang and Wang (2023) further highlighted that the interaction between motivation and self-regulation significantly predicts machine translation post-editing performance. However, such integrated perspectives remain rare, and most research continues to treat these factors separately. Therefore, this study seeks to address this gap by providing quantitative and qualitative evidence on how motivation and self-regulation interact to influence translation performance. By doing so, it aims to a more comprehensive understanding of cognitive and affective factors in translator training and offers practical insights for designing pedagogies that integrate both motivation and self-regulated learning strategies.

Drawing on previous findings, this study conceptualizes translation competence as a multidimensional construct influenced by both motivation and self-regulation. Prior research has shown that while motivation serves as an essential driver of learning engagement (Dörnyei and Ushioda, 2021; Nguyen et al., 2022), self-regulation enables students to strategically manage complex translation tasks (Zimmerman, 2002; Pietrzak, 2018). Studies such as Wen et al. (2023) have further refined this understanding by identifying specific self-regulatory strategies that predict translation performance. These insights provide a robust conceptual foundation for this study’s examination of the interplay between motivation, self-regulation, and translation competence in flipped classroom environments.

Taking the above considerations into account, this study aims to address the following four research questions to accomplish the objectives of the research:

*RQ1*: What is the impact of students’ motivation on their translation competence in the flipped translation classroom context?

*RQ2*: What is the impact of students’ self-regulation on their translation competence in the flipped translation classroom context?

*RQ3*: Which one of the following, namely, motivation or self-regulation, is a better predictor of students’ translation competence in the flipped translation classroom context?

*RQ4*: How do students’ perceptions of the flipped translation classroom relate to their motivation and self-regulated learning behaviors?

To guide the statistical analysis of the quantitative component, the following research hypotheses were formulated based on existing literature:

*H1*: Motivation has a significantly positive impact on translation competence in the flipped translation classroom context.

*H2*: Self-regulation has a significantly positive impact on translation competence in the flipped translation classroom context.

*H3*: Self-regulation is a stronger predictor of translation competence than motivation in the flipped translation classroom context.

## 3 Methodology

Ethical approval was obtained from the Research Ethics Committee of the university prior to commencement of data collection. Participants were provided with an information sheet detailing the study’s objectives, procedures, confidentiality safeguards, and their right to withdraw at any point without penalty. Written informed consent was obtained from all participants.

### 3.1 Participants

All 137 third-year undergraduate students majoring in Translation at a Chinese university constituted the total population and were included in the study. These students had completed the majority of core modules in translation studies and were engaged in a flipped classroom curriculum, making them a suitable group for investigating the research questions. In the curriculum design of this program, students spend the first 2 years primarily undertaking intensive language training courses to build linguistic proficiency, while specialized translation courses formally begin in the third year. Therefore, third-year students were selected as the most appropriate cohort for this research, as they possess both the language foundation and initial exposure to professional translation instruction necessary for investigating translation competence in a flipped classroom environment. In addition, third-year students were selected as participants due to their prior exposure to conventional classroom teaching during the first 2 years of study. This prior experience served as a baseline for comparison with the flipped classroom approach. What is more, all participants had achieved a high level of English proficiency, as evidenced by their college entrance examination scores exceeding 110 out of 150. The demographic composition included 21 males (16.03%) and 110 females (83.97%), reflecting a typical gender distribution in translation programs, as noted by Pym (2011) and Ameri and Ghahari (2018).

Prior to the main study, a pilot study was conducted with 30 students from this population to validate instruments and refine the research design. The remaining 137 students constituted the eligible population for the main study. Out of the 137 distributed questionnaires, 6 were excluded from the analysis due to respondents uniformly selecting the midpoint option (3: Neither Agree nor Disagree) on almost all items of the 5-point Likert Scale. This response pattern was interpreted as indicative of a lack of engagement with the survey content or an indecisive stance towards the questions posed. Additionally, questionnaires completed in less than 60 s were considered invalid based on the assumption that such a short completion time does not allow for thoughtful consideration of the questions, further justifying their exclusion. After applying these criteria, a total of 131 questionnaires were deemed valid for analysis, resulting in a valid response rate of 95.62%. As the study aimed to capture comprehensive data from this specific cohort, a census sampling strategy was employed rather than random sampling. The choice of census sampling aligns with the objective of achieving full coverage of the relevant student cohort, minimizing sampling bias. According to Cohen et al. (2018) census sampling is appropriate in educational research when the population is small, accessible, and clearly defined, as it enhances internal validity and reduces sampling error. While this method does not involve randomization, it remains acceptable for inferential analysis--such as correlation or regression--when findings are interpreted within the specific context of the group studied rather than generalized beyond it. As Gorard (2003) notes, the key lies in clearly defining sample boundaries and avoiding overgeneralization, thereby preserving the validity of statistical procedures within bounded populations. Given that the entire population of the current study was relatively small and fully accessible, census sampling offered a comprehensive view of the variables under investigation. We acknowledge, however, that as this is a near-complete census rather than a random sample, the reported inferential statistics should be interpreted with caution and understood as describing trends within this particular group of translation students.

### 3.2 Instruments

The research instruments utilized in this study included a comprehensive questionnaire of five-point Likert scales, semi-structured interviews, and translation tests. These tools were carefully selected to measure the core constructs of self-regulation, motivation, and translation competence, and to provide a multidimensional understanding of the students’ learning processes and outcomes in a flipped classroom setting. Modifications to the instruments were guided by feedback from a pilot study to ensure contextual relevance and validity.

#### 3.2.1 Reliability analysis

Before conducting further statistical analyses, it was crucial to evaluate the reliability of the measurement instruments used in this study. Since this research examines the interplay between self-regulation, motivation, and translation competence, ensuring that these constructs are measured with high internal consistency is fundamental for obtaining valid and interpretable results.

Cronbach’s Alpha coefficients were calculated for the three key scales—Motivation, Self-Regulation, and Translation Competence—to determine the reliability of the items measuring each construct. As shown in Table 1, all three scales demonstrated high internal consistency, with *α* values exceeding the recommended threshold of 0.80 (Nunnally, 1978). Among the three scales, the Self-Regulation Scale exhibited the highest reliability (*α* = 0.91), reflecting the well-defined nature of self-regulated learning strategies in translation tasks. This finding supports previous studies (e.g., Zimmerman and Kitsantas, 2014), which highlight that self-regulation is a multidimensional yet coherent construct that plays a critical role in learning complex cognitive skills such as translation. The Translation Competence Scale (*α* = 0.92) also demonstrated strong internal consistency, indicating that the measured dimensions—language-cultural competence, translator competence, and translation service provision competence—are well-aligned with existing models of translation competence (Beeby et al, 2011; Kiraly, 2000). The Motivation Scale (*α* = 0.89) showed similarly robust reliability, reinforcing the theoretical validity of motivation as a driving force in translation learning (Dörnyei and Ushioda, 2021). However, as will be discussed in subsequent sections, motivation alone may not suffice in predicting translation competence unless coupled with effective self-regulatory strategies.

**Table 1:** Correlation matrix and reliability test.

Variable	Motivation	Self-regulation	Translation competence	Number of items	Cronbach’s alpha (*α*)
Motivation	1	0.62**	0.59**	26	0.89
Self-regulation	0.62**	1	0.63**	24	0.91
Translation Competence	0.59**	0.63**	1	18	0.92

**p < 0.01.

These high reliability scores confirm that the instruments used in this study are suitable for assessing the relationships between self-regulation, motivation, and translation competence, thus strengthening the credibility of the subsequent statistical findings.

#### 3.2.2 Questionnaire

The questionnaire incorporated three scales: the Self-Regulation Scale, the Motivation Scale, and the Translation Competence Scale. Each scale was based on established instruments and adapted to suit the educational context of this study. All items were measured using a five-point Likert scale ranging from 1 (strongly disagree) to 5 (strongly agree).

##### 3.2.2.1 Self-regulation scale

The Self-Regulation Scale was adapted from the Online Self-Regulated Learning Questionnaire (OSLQ) developed by Barnard et al. (2009). The original scale comprises 24 items across six subscales: environment structuring, goal setting, time management, help-seeking, task strategies, and self-evaluation. Modifications were made to align with translation-specific tasks in the flipped classroom. For example, items on environment structuring were tailored to reflect the preparation of translation projects, and task strategies were adjusted to emphasize cognitive strategies specific to translation. The revised scale retained all 24 items, ensuring coverage of key self-regulation dimensions. The decision to retain all 24 items in the Self-Regulation Scale was based on a rigorous validation process. The items were reviewed by experts to ensure cultural and linguistic relevance. Exploratory factor analysis showed strong factor loadings for all items and excellent construct validity, while reliability analysis confirmed high internal consistency (Cronbach’s alpha > 0.90). Therefore, no items required deletion and the full set of 24 items was retained as they all contributed meaningfully to the measurement of self-regulation in this context.

##### 3.2.2.2 Motivation scale

The motivation scale used in this study was adapted from “Motivation Scales” subscale of the Motivated Strategies for Learning Questionnaire (MSLQ), developed by Pintrich et al. (1991). The MSLQ motivation scale was chosen because it provides a comprehensive and validated framework that captures both intrinsic and extrinsic motivational dimensions, task value, and self-efficacy—factors highly relevant to translation learning in a flipped classroom. Grounded in the social-cognitive framework of motivation, MSLQ is a widely recognized tool for its established psychometric properties, adaptability, and prior successful use in L2 and academic learning contexts, making it an appropriate for this study’s aims. The original scale comprised 31 items spanning six subscales: intrinsic goal orientation, extrinsic goal orientation, task value, control of learning beliefs, self-efficacy for learning and performance, and test anxiety.

For this study, the scale was modified to align with the flipped translation classroom context. Following a pilot study and feedback from experts and participants, the revised version retained 26 items to better reflect the attitudes, behaviors, and beliefs of translation-major students in China. Specific adaptations included contextualizing intrinsic and extrinsic goal orientations to translation tasks and refining task value items to emphasize the practical relevance of translation skills. These modifications ensured the scale’s relevance and reliability in measuring the motivational constructs within the educational setting of translation training.

##### 3.2.2.3 Translation competence scale

The Translation Competence Scale was adapted from the model proposed by Fatma and Çoban, 2022, encompassing 21 items of three sub-competences of: language-cultural competence, translator competence, and translation service provision competence. A pilot study was conducted to examine the psychometric properties of this scale. Exploratory factor analysis (EFA) was performed, and items with factor loadings below 0.4 or low corrected item-total correlations (<0.3) were deleted. Consequently, three items were removed, resulting in an 18-item scale with a clear factor structure and high internal consistency (Cronbach’s alpha > 0.90). This process ensured that all retained items reliably measured key dimensions of translation competence within this context. The scale was refined to include 18 items across three dimensions: language-cultural competence (7 items), translator competence (6 items), and translation service provision competence (5 items). These dimensions were designed to capture the multifaceted nature of translation competence within educational contexts.

#### 3.2.3 Semi-structured interviews

To complement the quantitative data, semi-structured interviews were conducted with a subset of participants. The interviews aimed to explore students’ perceptions of the flipped classroom model and their strategies for self-regulation and motivation. These qualitative insights enriched the study by contextualizing the quantitative findings within the participants’ lived experiences.

The interview guide was meticulously developed based on key themes identified from the literature, ensuring alignment with the study’s objectives and theoretical framework (Patton, 2014; Adams, 2015). The guide comprised an agenda, core open-ended questions, and prompts for probing deeper responses. The thematic structure included: (1) perceived benefits and challenges of flipped learning; (2) strategies students used for self-regulation; (3) factors influencing motivation to engage in flipped classroom activities.

To ensure the clarity and appropriateness of the questionnaire, three experts in educational psychology, translation studies, and second language acquisition were invited to review the instrument. They assessed the linguistic clarity, content relevance, and cultural appropriateness of all items. Based on their suggestions, minor adjustments were made to the wording of several items to enhance clarity and contextual suitability.

Subsequently, a pilot study was conducted with 30 third-year translation undergraduates (excluded from the main sample). Participants provided feedback on the clarity and comprehensibility of the items. Based on this feedback, several items were further revised to improve readability and ensure that the instrument was well-aligned with the participants’ academic context and cognitive level. The final interviews were conducted individually, face-to-face, in a quiet, comfortable environment conducive to reflection. Each interview lasted approximately 90 min, allowing time for detailed discussion, clarifications, and observation of non-verbal cues (Rolland et al., 2019). All interviews were recorded with participants’ consent and transcribed verbally for thematic analysis. All transcripts were anonymized to protect participants’ confidentiality.

Interview participants were purposively sampled from the larger cohort based on their quantitative translation competence scores to ensure representation across performance levels: eight students of medium competence and two students each from the highest and lowest quartiles.

### 3.3 Data analysis

**Figure 1:**
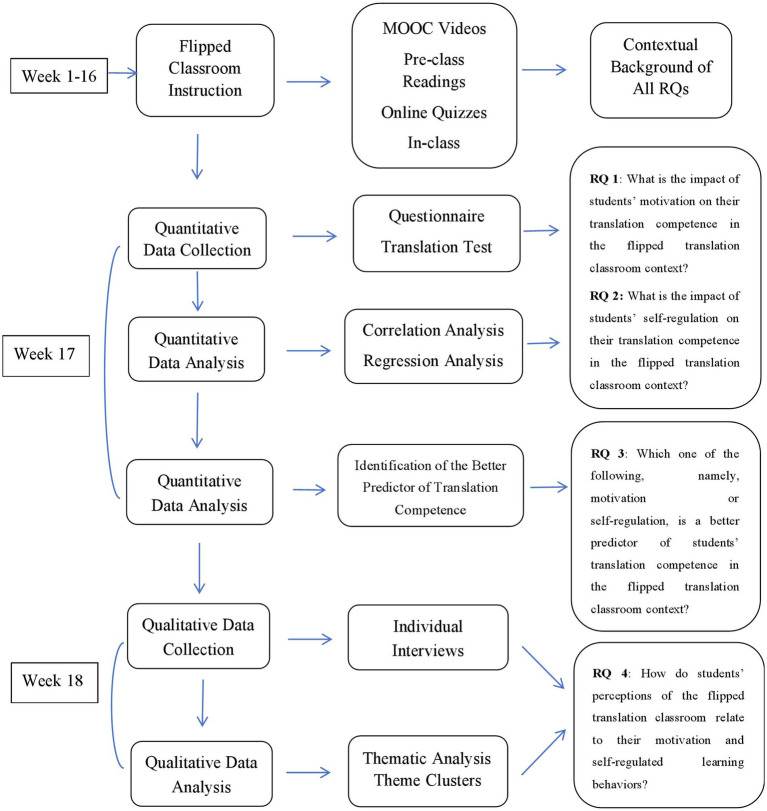
Instructional design and data collection flowchart aligned with RQs.

The flipped classroom intervention spanned a full 16-week semester and was structured using the MOOC-based online platform. Before each in-class session, students were required to complete online preparatory activities, including viewing video lectures, reading instructor-prepared materials, and participating in online discussions on the MOOC forum. In-class sessions focused on collaborative translation exercises, quizzes, role plays, and peer feedback. This design aimed to foster autonomy, enhance engagement, and provide opportunities for students to apply theoretical knowledge in practical tasks. A visual summary of the instructional design and data collection timeline aligned with the study’s research questions is presented in Figure 1.

Data collection was systematically organized as follows: during Weeks 1–16, students engaged in the flipped classroom activities while formative data (e.g., forum participation) were monitored. Immediately after course completion (Week 17), questionnaires and translation tests were administered. In Week 18, semi-structured interviews were conducted with purposively selected students to gather qualitative insights contextualizing the quantitative findings.

Data analysis in this study employed a mixed-methods approach to ensure a comprehensive understanding of the research questions. Quantitative data from the questionnaires and translation tests were analyzed using statistical methods, with regression analysis serving as the primary technique to evaluate the relationships between motivation, self-regulation, and translation competence. This analysis identified the predictive roles of motivational and self-regulatory strategies on translation performance, providing insights into the relative importance of each factor. Descriptive statistics, such as means, standard deviations, and frequency distributions, were used to summarize the overall trends in the data.

Qualitative data obtained from the interviews were analyzed using thematic analysis, following Braun and Clarke’s (2019) six-phase framework. This involved transcribing the interview recordings, coding the data, and identifying recurring themes. Themes were refined and categorized to capture participants’ perceptions of the flipped classroom, the challenges they faced, and the strategies they employed to maintain motivation and regulate their learning. The use of NVivo software facilitated the organization and management of qualitative data, ensuring a systematic and thorough analysis. The software was then used to organize these codes into categories by facilitating keyword searches, clustering similar codes, and refining categories through iterative comparison. This process ensured consistency and traceability in theme development while enhancing the rigor of qualitative analysis. Inter-coder reliability was enhanced by involving two additional independent coders who reviewed a transcript and discussed discrepancies, leading to consensual definitions of codes and categories.

By integrating quantitative and qualitative data, the study provided a holistic view of the complex interplay between motivation, self-regulation, and translation competence in a flipped classroom setting. This mixed-methods approach allowed the findings to address both measurable outcomes and nuanced experiences, contributing to a more comprehensive understanding of the research problem.

## 4 Results and findings

### 4.1 Descriptive and correlation analysis

To gain a clearer understanding of the relationships among translation competence, self-regulation, and motivation, descriptive statistics and correlation analyses were conducted. These analyses provide valuable insights into how students’ ability to regulate their learning and their motivational levels interact with their translation performance.

Table 1 presents the correlation matrix for the three key variables. The results reveal a strong positive correlation between self-regulation and translation competence (r = 0.63, *p <* 0.01), suggesting that students who effectively plan, monitor, and reflect on their learning tend to achieve higher translation proficiency. Notably, self-regulation exerts a stronger influence than motivation (r = 0.59, *p <* 0.01), reinforcing the idea that successful translators are not merely driven by motivation but also by the ability to strategically manage their learning processes (Zimmerman, 2000).

These findings align with previous research in translation studies and self-regulated learning. Studies by Hu et al. (2021) and Pietrzak (2018) similarly emphasized that self-regulated learners are more adept at navigating the complexities of translation tasks, as they employ structured cognitive strategies to tackle challenges. Consistent with these findings, Barakat (2024) reported that applying self-regulation strategies not only improved translation skills but also positively shaped students’ attitudes toward translation learning. Similarly, Bolaños-Medina and Núñez (2022) highlighted that autonomy support, critical thinking, and motivation jointly predict strategic translation competence, underlining the importance of self-regulated behaviors. Moreover, Li et al. (2023) demonstrated that self-efficacy, a key aspect of self-regulation, mediates the relationship between critical thinking and translation technology competence, further reinforcing the intertwined nature of these constructs. Moreover, the significant yet slightly lower correlation between motivation and translation competence suggests that while motivation remains an important factor, it may serve as an indirect facilitator rather than a primary determinant of competence, as also implied in these recent studies.

### 4.2 The impact of motivation on translation competence

The following section explores how students’ motivation influences their translation competence in the flipped classroom setting, thereby addressing the first research question. Motivation has long been recognized as a key factor in second language acquisition and translation learning, driving students’ engagement and persistence (Dörnyei and Ushioda, 2021). However, its direct impact on translation competence remains a subject of debate, particularly in contexts where translation tasks demand sustained cognitive effort and strategic regulation. This study sought to clarify the role of motivation by examining its predictive power in determining students’ translation competence.

A regression analysis was conducted to assess the extent to which motivation influences translation competence. As shown in Table 2, motivation significantly predicted translation competence, accounting for 34.3% of the variance (R^2^ = 0.343, *F* = 67.394, *p <* 0.001). The standardized regression coefficient (*β* = 0.586, *p <* 0.001) indicates a moderate-to-strong positive effect, confirming that students with higher motivation levels tend to perform better in translation tasks.

**Table 2:** The impact of motivation and self-regulation on translation competence.

Variables	Variables and dimensions	Beta	t	p
Motivation	Learning Motivation (Overall)	0.586	8.209	<0.001
	Intrinsic Goal Orientation	0.215	2.906	<0.001
	Extrinsic Goal Orientation	0.205	2.917	<0.001
	Task Value	0.231	2.808	<0.001
	Control of Learning Beliefs	0.310	2.537	<0.001
	Self-Efficacy for Learning and Performance	0.273	2.715	<0.001
	Test Anxiety	0.284	2.682	<0.001
Self-Regulation	Self-Regulated Learning (Overall)	0.628	9.167	<0.001
	Goal-setting	0.454	4.048	<0.001
	Environment Structuring	0.262	3.762	<0.001
	Task Strategies	0.282	4.132	<0.001
	Time Management	0.295	4.224	<0.001
	Help-seeking	0.250	3.509	<0.001
	Self-evaluation	0.396	5.146	<0.001

However, while motivation plays a substantial role, its influence must be interpreted within the broader context of self-regulated learning. Motivation alone does not guarantee effective translation performance unless accompanied by well-developed self-regulation skills (Zimmerman, 2000). This aligns with Ameri and Sadrnia (2025) findings, which suggest that intrinsically motivated students exhibit greater persistence and creativity in translation tasks, yet may struggle if they lack structured learning strategies.

Further analysis of the sub-dimensions of motivation (Table 2) revealed that Control of Learning Beliefs (*β* = 0.310, *p <* 0.001) exerted the strongest influence on translation competence. This suggests that students who perceive themselves as in control of their learning outcomes are more likely to excel in translation tasks, reinforcing the argument that motivation must be accompanied by cognitive engagement and strategic planning (Liu and Yu, 2019).

Additionally, Task Value (*β* = 0.231, *p <* 0.001) and Intrinsic Goal Orientation (*β* = 0.215, *p <* 0.001) were significant predictors, highlighting that students who see translation as a meaningful and valuable skill are more engaged and perform better. This aligns with Pym et al.’s (2013) view that translation education must emphasize the relevance of translation tasks to real-world professional settings to sustain student motivation.

In contrast, Test Anxiety (*β* = 0.284, *p <* 0.001) also emerged as a notable predictor, suggesting that high-pressure academic environments may negatively impact translation performance by inducing cognitive overload. This finding underscores the importance of creating a balanced learning environment where motivation is nurtured without excessive pressure.

Overall, these results confirm that motivation is an important but insufficient predictor of translation competence. While motivated students demonstrate higher engagement and task persistence, they may still struggle with complex translation challenges if they lack self-regulation skills—a point that will be further explored in the next section.

### 4.3 The impact of self-regulation on translation competence

While the previous section established motivation as a key driver of engagement in translation learning, motivation alone does not fully explain variations in translation competence. This section explores how students’ self-regulated learning correlates with their translation competence, thereby addressing the second research question. Research in self-regulated learning (SRL) suggests that students must actively plan, monitor, and regulate their learning processes to achieve high performance (Zimmerman, 2000). Given the complexity of translation tasks—requiring linguistic, cognitive, and metacognitive coordination—self-regulation is expected to play a crucial role in developing translation competence.

To assess the predictive power of self-regulation, a regression analysis was conducted (see Table 2). The standardized regression coefficient (*β* = 0.628, *p <* 0.001) suggests that students who exhibit strong self-regulation skills consistently perform better in translation tasks. This finding reinforces the argument that translation is a cognitively demanding activity that requires strategic learning behaviors (Shreve, 2017).

Notably, the predictive strength of self-regulation (*β* = 0.628) surpasses that of motivation (*β* = 0.586, see Table 3), indicating that while motivation initiates learning engagement, self-regulation sustains and enhances performance. This aligns with previous research in translator training (e.g., Pietrzak, 2018; Hu et al., 2021), which emphasizes that students who actively regulate their learning tend to demonstrate higher accuracy, problem-solving abilities, and adaptability in translation tasks.

**Table 3:** Regression results of motivation and self-regulated learning on translation competence.

Variables	Non-standardized coefficients		Standardization coefficient	*t*	*p*	Collinearity diagnostics	
	*B*	Standard error	*Beta*			Tolerance	VIF
Constant	0.494	0.344	–	1.434	0.154	–	–
Motivation	0.385	0.120	0.293	3.214	0.002**	0.528	1.894
Self-regulated learning	0.467	0.100	**0.427**	4.692	0.000**	0.528	1.894
*R* ^2^	0.440						
Adjust *R*^2^	0.431						
*F*	*F* (2,128) = 50.211, *p* = 0.000						

Dependent variable: translation competence.

*p < 0.05, **p < 0.01.

The bold value indicates the stronger predictor of the dependent variable.

Further analysis of the sub-dimensions of self-regulation (see Table 2) provides deeper insights into which specific strategies contribute most to translation competence. Goal-setting (*β* = 0.454, *p <* 0.001) and self-evaluation (*β* = 0.396, *p <* 0.001) emerged as the strongest predictors, highlighting the importance of deliberate planning and reflective learning.

Goal-setting enables students to prioritize translation challenges, break down complex tasks into manageable steps, and maintain a clear learning trajectory. This supports previous findings by Zimmerman and Kitsantas (2014), who argue that goal-oriented learners are more likely to engage in deep learning strategies and sustain their efforts over time. Qualitative data also reinforce this point, as one participant (P7) noted:

*Setting clear translation goals helps me stay focused. If I do not plan my learning, I tend to procrastinate and struggle with long texts.*

Similarly, self-evaluation plays a critical role in helping students identify errors, refine their translation strategies, and track their progress over time. Participants who engaged in frequent self-reflection demonstrated higher levels of accuracy and consistency in translation tasks. As P3 shared:

*I often review my past translation mistakes and try to understand what went wrong. This really helps me avoid making the same errors in future tasks.*

Beyond these key factors, time management (*β* = 0.295, *p <* 0.001) and task strategies (*β* = 0.282, *p <* 0.001) also contributed significantly to translation competence. Effective time management allows students to allocate sufficient time for different stages of the translation process (e.g., pre-reading, drafting, revision), whereas structured task strategies help them navigate complex texts with greater efficiency.

These findings suggest important pedagogical implications for translator training programs. Traditional translation courses often focus heavily on linguistic accuracy and textual fidelity, but fail to systematically cultivate self-regulation strategies. Given that self-regulation plays a more dominant role than motivation, it is essential for translation educators to integrate explicit SRL training into curricula. Practical interventions—such as guided goal-setting exercises, reflective translation journals, and structured self-assessment tools—can equip students with the metacognitive skills necessary for long-term professional success.

Overall, the results confirm that self-regulation is an influential determinant of translation competence, reinforcing the idea that successful translators are not merely motivated learners, but also strategic and self-directed ones.

### 4.4 Identifying the better predictor of translation competence

This section examines the predictive strengths of motivation and self-regulation on translation competence through multiple regression analysis, in response to the third research question. The findings have demonstrated that both motivation and self-regulation significantly influence translation competence. However, given their distinct roles in learning, it remains crucial to determine which factor serves as the stronger predictor of translation success. This distinction is particularly important for translation pedagogy, as it informs educators about which skills to prioritize in curriculum design.

To address this, a comparative regression analysis was conducted (see Table 3). The results indicate that self-regulation (*β* = 0.427, *p <* 0.001) remains the dominant predictor of translation competence, whereas motivation (*β* = 0.293, *p* = 0.002) plays a comparatively weaker role. This confirms that, while motivation is essential for initiating learning, sustained translation competence development relies more heavily on self-regulation strategies (Zimmerman, 2002).

This argument is further supported by qualitative data from student interviews. Many students expressed that, although they were initially motivated to improve their translation skills, they often felt overwhelmed when faced with complex texts. As P6 noted:

*“I always start with strong motivation, but when I encounter a difficult passage, I sometimes do not know how to tackle it. That’s when I realize that having a clear strategy is even more important than just wanting to do well.”*

Another participant (P9) reinforced this idea:

*“Motivation helps me get started, but without a structured plan, I find myself struggling to complete the task effectively. When I actively reflect on my mistakes and adjust my strategies, I see real improvement.”*

These accounts illustrate that while participants generally began with high motivation, some encountered difficulties when facing complex translation tasks. For example, P6 reported that motivation alone was not enough to complete challenging passages effectively, while P9 described struggling to apply appropriate strategies when lacking a clear plan. These responses reflect varied student experiences in managing translation tasks within the flipped classroom.

### 4.5 Qualitative results

The final research question is addressed through thematic analysis of qualitative data collected from 12 participants (P1–P12), focusing on their perceptions of the flipped translation classroom and how these relate to their motivational and self-regulatory behaviors. Consistent with qualitative research practice, 12 participants (approximately 9% of the full sample of 137) were selected using purposive sampling based on their willingness and ability to provide rich data forinterviews. This proportion aligns with recommendations in mixed-methods research for selecting about 10% of the larger sample for qualitative inquiry (Onwuegbuzie and Collins, 2007), while also conforming to Creswell’s (2015) suggestion that 10–15 participants are sufficient to reach data saturation in qualitative studies. Thematic analysis following Braun and Clarke’s methodology identified three key themes: challenges in learning, teacher-student interaction, and mixed attitudes toward the flipped model (see Table 4).

**Table 4:** Students’ perceptions of the flipped translation classroom.

Themes	Key findings
Challenges in learning	Difficulty integrating online and offline sessions; forgetting online content; note-taking struggles.
Teacher-student interaction	Lack of immediate support; missing classroom atmosphere; need for teacher guidance.
Mixed attitudes	Flexibility in pacing learning; preference for traditional instruction; reluctance to recommend FC.

#### 4.5.1 Challenges in learning

The thematic analysis of participant responses revealed two prominent challenges in learning within the flipped translation classroom: difficulty integrating pre-class online materials into in-class sessions and struggles with note-taking during online learning. These difficulties are indicative of limitations in students’ self-regulatory capacities, such as planning, preparation, and self-monitoring.

For example, participant P5 noted that they sometimes did not listen to the relevant material before class, leading to forgetfulness during in-class discussions and difficulty connecting learning across phases—a key aspect of effective self-regulation. This was echoed by P8, who stated, “*If the teacher does not review online content in class, I struggle to connect the two,*” highlighting the challenge of transferring knowledge from independent preparation to classroom application.

Forgetfulness was another recurring issue illustrating weak self-monitoring, as P2 reflected, “*I hear the material online, but by the time we are in class, I’ve forgotten it.*” Such gaps reduced knowledge retention and application, reinforcing the importance of sustained self-regulatory efforts. Additionally, students reported note-taking difficulties when engaging with lengthy and complex online materials. As P9 shared, “*I cannot adjust the video easily, so I miss key points when taking notes,*” reflecting cognitive overload and a lack of effective learning strategies.

While these students may have expressed motivation to succeed, their reported experiences suggest that motivation alone was insufficient to overcome the demands of the flipped classroom without robust self-regulation strategies. These qualitative insights thus complement the quantitative findings, further supporting the conclusion that self-regulation plays a more critical role than motivation in predicting translation competence.

#### 4.5.2 Teacher-student interaction

Teacher-student interaction emerged as a recurring theme in the interview transcripts, with many students emphasizing the pivotal role of teachers in providing support. Many participants emphasized the pivotal role of teacher support in their learning, with its absence in online sessions posing challenges for engagement and comprehension. For example, P1 remarked, “*It (online session) rarely resembles the atmosphere of our offline classes, such as the way our teachers speak to us more intimately about certain topics, which is very important. We really missed the good atmosphere.*” This sentiment illustrates that even motivated learners may feel disconnected and struggle without teacher facilitation, reinforcing that motivation alone does not suffice without a supportive structure to guide learning.

Similarly, P4 shared, “*I feel like we need help from the teacher.when the teacher asks us questions during class and organizes it, I feel like there’s a sudden awakening,*” highlighting that teacher guidance promotes deeper understanding—a key component of effective self-regulation. This underscores that while flipped classrooms aim to foster learner autonomy, teacher intervention remains essential in scaffolding students’ metacognitive processes such as reflection and comprehension monitoring.

The absence of immediate feedback also impacted students’ confidence in their translation competence. As P1 further noted, “*Because translation itself is quite flexible. we did not know whether the word combinations are correct, or if the words I choose are suitable,*” reflecting uncertainty that can hinder both motivation and performance without expert feedback. Likewise, P9 observed, “*Combining online and offline aspects. presents certain difficulties. perhaps requiring assistance from teachers,*” illustrating the need for instructional support to help students navigate complex translation tasks and transfer knowledge between online preparation and in-class application.

Overall, these qualitative insights reveal that effective teacher-student interaction is not merely a preference but integral to fostering translation competence in the flipped classroom. Teachers facilitate critical self-regulation processes—clarifying ambiguity, providing feedback, and maintaining engagement—thereby complementing students’ intrinsic motivation and supporting their ability to manage their own learning effectively.

#### 4.5.3 Mixed attitudes toward the flipped classroom

While some students appreciated the flexibility of the flipped model, others were hesitant about its effectiveness. The feedback from participants reflected complex attitudes toward the flipped classroom. Seven out of twelve respondents expressed clearly positive attitudes and indicated that they would recommend this mode of learning to others. For instance, P1 stated, “*I would recommend the flipped translation course to other translation learners or lovers because teachers summarize the key points in the videos very clearly in the online session and the schedule organization is very clear.*” Similarly, P7 appreciated the flipped approach for providing flexibility, noting that “*the flipped classroom teaching allowed her to have some time to rest and reflect on the learning process.*”

At the same time, four out of twelve participants demonstrated mixed or ambivalent views, acknowledging certain benefits but highlighting challenges. For example, P1 mentioned the convenience of self-paced learning: “*I can pause or skip content when needed, which is convenient.*” This flexibility helped reduce anxiety and allowed students to revisit difficult material. However, participant P8 noted, “*Most students still prefer traditional classes over online learning*,” and although P8 held a generally positive attitude, they explicitly declined to recommend the flipped model to others, reflecting students’ complex attitudes towards the flipped classroom.

This pattern of responses reveals that while the flipped classroom offers benefits such as flexibility and self-pacing, some students struggled with maintaining engagement in a less structured, non-monitored environment. Therefore, the theme “Mixed Attitudes” was identified to capture this complexity: even participants who recognized the advantages of flipped learning sometimes hesitated to fully endorse it due to perceived limitations in interaction and supervision.

### 4.6 Integration of quantitative and qualitative findings

The integration of quantitative and qualitative findings indicates the dominant role of self-regulation in the development of translation competence. Quantitative analysis identified self-regulation as the most significant predictor, accounting for 39.4% of the variance, surpassing motivation in its impact. The qualitative data further reinforced this conclusion by illustrating the ways in which effective self-regulatory strategies contribute to translation performance.

The qualitative findings align with the quantitative results, demonstrating that individuals who establish clear learning objectives, monitor their progress, and adjust their strategies achieve superior translation outcomes. This corresponds with the statistical significance of goal-setting (*β* = 0.454, *p <* 0.001) and self-evaluation (*β* = 0.396, *p <* 0.001), identified as the most influential predictors. Additionally, structured time management (*β* = 0.295, *p <* 0.001) and task strategies (*β* = 0.282, *p <* 0.001) were crucial for effective translation learning, reinforcing the importance of systematic study planning.

In contrast, individuals lacking self-regulatory habits exhibited challenges in sustaining translation performance, despite high motivation levels. These findings highlight that motivation alone is insufficient to enhance translation competence without the support of structured self-regulation. While motivation demonstrated a positive association with translation competence (*β* = 0.586, *p <* 0.001), qualitative insights suggest that its impact is inconsistent. Some learners benefited from the autonomy of the flipped classroom model but encountered difficulties in maintaining direction without structured guidance. This suggests that motivation, while valuable, does not directly translate into competence unless reinforced by self-regulated learning strategies.

The convergence of quantitative and qualitative evidence affirms that self-regulation is the primary driver of translation competence. Quantitative data provide empirical validation, while qualitative findings offer deeper insights into the mechanisms by which self-regulation facilitates learning. These results emphasize the need for translation education to prioritize structured self-regulation training, positioning motivation as a complementary factor rather than the central mechanism for skill development.

## 5 Discussion

The findings of this study reveal a compelling interplay between motivation, self-regulation, and translation competence, shedding light on how students navigate the complexities of translation learning. While motivation is often regarded as a key driver of engagement (Deci and Ryan, 2013), this study provides empirical evidence that motivation alone is insufficient; students must develop effective self-regulation strategies to achieve sustained success in translation tasks.

### 5.1 The impact of motivation on translation competence

In response to Research Question 1, which explores the impact of motivation on students’ translation competence within the flipped translation classroom, this section discusses the empirical findings derived from the quantitative data analysis. This study found that motivation exerts a positive influence on students’ translation competence, a result that aligns with prior research in general educational settings (Ameri and Ghahari, 2018; Tseng and Yeh, 2019; Yusuf, 2011). Grounded in self-determination theory, motivation in this study was conceptualized as a multidimensional construct that interacts dynamically with learning processes in the context of translation pedagogy.

However, this finding diverges from the results reported by Yang and Wang (2023), who found no significant impact of motivation on students’ performance in machine translation post-editing (MTPE) tasks. This discrepancy may be attributed to contextual and pedagogical differences. Specifically, MTPE remains a relatively novel practice in the Chinese translation curriculum, and its limited integration may hinder students’ engagement with such tasks. Additionally, skepticism toward machine translation among educators—who may perceive it as a shortcut rather than a learning tool—could further reduce students’ motivation to invest cognitively in MTPE activities (Van Praag and Sanchez, 2015; Yang and Wang, 2023). As Briggs (2018) and Huang et al. (2020) note, the cognitive demand in MTPE is typically lower than in conventional translation tasks, potentially weakening the observable connection between motivation and learning outcomes.

By contrast, the current study involved conventional written translation tasks, which required deeper cognitive engagement—including processes such as paraphrasing, addition, and omission (Niño, 2008)—and were closely aligned with students’ prior training and perceived professional relevance. This alignment between the task demands and students’ learning experiences may have strengthened the motivational effect observed in the present study. These findings lend empirical support to Hypothesis 1 (H1), which posited that motivation has a positive impact on translation competence.

### 5.2 The impact of self-regulation on translation competence

In response to RQ2, which examines the impact of self-regulation on students’ translation competence, the quantitative findings from this study demonstrate that self-regulated learning (SRL) significantly predicts translation competence within a flipped classroom environment. This aligns with recent empirical evidence emphasizing SRL’s critical role in translation education. For example, Wen et al. (2023) developed and validated the Self-Regulated Translation Learning Strategy Scale (SRTLSS), confirming robust predictive validity among EFL learners. Yang and Wang (2023) further identified SRL as a key predictor of machine translation post-editing performance. Additionally, Barakat (2024) showed that direct instruction in self-regulation strategies significantly enhances translation skills and positive attitudes towards translation tasks.

Within our study, goal-setting and self-evaluation emerged as the most influential SRL dimensions (accounting for R^2^ ≈ 28%), followed by task strategies and time management. The strong effects of planning and reflective evaluation corroborate SRL theory and the empirical findings of post-testing interventions in translation training. These results underscore the pedagogical importance of embedding SRL strategy instruction into translator training curricula to foster both translation performance and learner autonomy. Taken together, the results confirm the centrality of SRL in translation learning and provide empirical support for Hypothesis 2 (H2), which posited a positive impact of self-regulation on translation competence.

### 5.3 Stronger predictor on translation competence

In addressing Research Question 3, this study hypothesized that self-regulation would serve as a stronger predictor of students’ translation competence than motivation (H3). The results of the multiple regression analysis confirmed this hypothesis, revealing that self-regulation explained a greater proportion of variance in translation competence compared to motivation. The data analysis demonstrated that self-regulation outperformed motivation as a predictor of translation competence. This is hardly surprising given the cognitive and metacognitive demands of translation. Unlike general language learning, which often relies on habitual exposure and reinforcement, translation requires deliberate decision-making, strategic planning, and problem-solving, all of which are core aspects of self-regulated learning (Zimmerman, 2002). A motivated student may eagerly approach a translation task, but without the ability to set realistic goals, monitor progress, and refine strategies, motivation can quickly fade in the face of challenges.

This finding aligns with studies in both translation studies and educational psychology. Pietrzak (2018) found that expert translators demonstrate significantly higher levels of self-regulation compared to novices, suggesting that translation competence is not merely a matter of linguistic proficiency but of strategic learning behavior. Similarly, Hu et al. (2021) emphasized that students who actively self-monitor their progress and reflect on errors tend to improve their translation accuracy over time, reinforcing the idea that self-regulation facilitates deeper engagement with the translation process.

Furthermore, recent research by Kreutzer and Riezler (2019) highlights the importance of self-regulation in interactive sequence-to-sequence learning, noting that self-regulated learners are more likely to achieve academic success and develop lifelong learning skills. This perspective is supported by Kormos and Csizér (2014), who argue that strong instrumental goals and international posture, along with positive future self-guides, are prerequisites for the use of effective self-regulatory strategies, which in turn influence autonomous learning behaviors.

Additionally, a study by Imhof et al. (2024) examined the role of self-regulated learning activities on academic competence gain in an emergency remote teaching context, finding that self-regulation is a critical factor for coping with the challenges of independent studying. This underscores the importance of fostering self-regulatory skills to enhance academic performance in various learning environments.

Yet, this study also highlights an important phenomenon: motivation and self-regulation are not mutually exclusive but closely related. In other words, motivation initiates learning engagement; self-regulation sustains and optimizes learning performance. While self-regulation plays a more dominant role than motivation, both contribute to translation competence in complementary ways.

Students with strong motivation but weak self-regulation may struggle with task persistence, whereas those with high self-regulation but low motivation may approach translation as a mechanical process rather than an intellectually engaging challenge. This suggests that while self-regulation plays a dominant role, motivation remains an essential catalyst, particularly in sustaining long-term learning efforts.

Given the crucial role of self-regulation, these findings call for a fundamental shift in how translation competence is cultivated in educational settings. Traditional translation pedagogy has often emphasized linguistic precision, textual analysis, and bilingual proficiency, assuming that students will develop effective learning strategies organically. However, this study suggests that explicit self-regulation training should be integrated into translator education to help students develop autonomy, adaptability, and cognitive flexibility.

These findings highlight the necessity of positioning self-regulation as a fundamental rather than auxiliary component of translation training. Many students enter translation programs without a structured approach to managing complex texts, highlighting the need to integrate goal-setting and strategic planning into translation curricula. By incorporating explicit goal-setting frameworks—such as breaking down assignments into clear, achievable milestones—students can develop greater autonomy over their learning trajectory. This is particularly relevant in a flipped classroom environment, where students must engage with materials independently before class sessions. Additionally, self-monitoring has emerged as a critical determinant of translation competence, yet many students lack systematic self-evaluation habits. Embedding reflective practices, such as self-assessment rubrics, translation journals, and peer feedback sessions, can foster metacognitive awareness and critical engagement with translation choices.

Another key consideration is the role of adaptive problem-solving in translation competence. Qualitative data suggest that students who struggle with translation challenges often lack the flexibility to adjust their strategies. Explicit strategy training—where students experiment with different translation approaches (e.g., literal vs. sense-for-sense) and evaluate their effectiveness—can cultivate metacognitive adaptability, a hallmark of skilled translators. These pedagogical interventions align with constructivist approaches to translator training (Kiraly, 2014), which emphasize active, student-centered learning experiences. By embedding self-regulation into translation pedagogy, educators can equip students not only with linguistic and technical competencies but also with the cognitive resilience necessary for professional success.

While this study reinforces the dominant role of self-regulation over motivation in predicting translation competence, it is important to position these findings within the wider field of learning research. For instance, Sun et al. (2023) conducted a comprehensive meta-analysis and found that flipped classroom environments significantly enhance student self-efficacy and engagement—both of which are closely linked to self-regulatory processes. Moreover, Xu et al. (2023) highlighted that self-regulated learning programs across online and blended contexts yield stronger academic outcomes than motivation-driven interventions. In contrast, mainstream SLA studies often foreground motivation (Dörnyei and Ushioda, 2021). This juxtaposition emphasizes that although motivation initiates engagement, complex translation tasks require sustained strategic control—that is, deeper self-regulation.

Furthermore, this study contributes important new insights to the flipped learning literature, particularly regarding the necessity of scaffolding metacognitive support. Judy Shih and Huang (2022) found that EFL learners demonstrate improved metacognitive strategy use when flipped classrooms explicitly incorporate metacognitive scaffolding. Similarly, Wang and Zhang (2024) demonstrated that collaborative online flipped environments significantly boost self-regulation and academic performance in language contexts. Our findings extend these results by showing that, unless learners are equipped with self-regulatory skills, increased motivation in flipped settings does not automatically translate into higher translation competence. This underscores a pedagogical imperative: translation curricula should complement flipped classroom models with structured instruction in goal-setting, monitoring, and adaptive revision strategies.

### 5.4 Linking perceptions with learning behaviors

In response to Research Question 4, which investigates how students’ perceptions of the flipped translation classroom relate to their motivation and self-regulated learning behaviors, the qualitative findings demonstrate a nuanced relationship. While most students expressed appreciation for the flexibility and autonomy afforded by flipped instruction—an observation aligned with Nguyen (2021) and Purwanto et al. (2022)—many also reported challenges such as forgetfulness, limited note-taking strategies, and insufficient teacher interaction, all of which impeded their self-regulated engagement. This ambivalence reflects a gap between learner autonomy and preparedness for self-directed learning. As Colomo-Magaña et al. (2020) argue, flipped classrooms are most effective when accompanied by explicit training in SRL strategies. Additionally, students’ desire for teacher support highlights an ongoing dependence shaped by traditional teacher-centered models (Hsu et al., 2019; Barakat, 2024). These findings suggest that while the flipped classroom holds motivational potential, its success relies on integrating metacognitive scaffolding to develop learners’ autonomous learning capacities.

## 6 Conclusion

The current study investigated the pivotal role of self-regulation in translation competence, demonstrating that motivation alone is insufficient for success in translation learning. While motivation remains a necessary component, it is self-regulation that ultimately determines whether students can navigate the complexities of translation tasks effectively.

### 6.1 Summary of findings

The quantitative findings revealed that self-regulation had a stronger positive correlation with translation competence than motivation, underscoring the centrality of self-regulatory behaviors—such as goal-setting, self-monitoring, and reflection—in translation learning. The qualitative analysis enriched this understanding by illustrating how students who actively employed self-regulation strategies, such as planning their study schedules and reflecting on their translation processes, reported greater confidence and competence. In contrast, students who primarily relied on initial motivation without sustained self-regulation expressed greater difficulty managing complex translation tasks. Moreover, interviews revealed that teacher-student interaction and structured guidance were perceived as crucial for supporting self-regulated learning, particularly in flipped classroom contexts where independent study predominated. Overall, these findings suggest that while motivation provides the initial impetus for engagement, it is the application of self-regulation strategies that enables students to translate effort into improved competence.

### 6.2 Implications

These findings have important implications for both theoretical models of translator training and practical classroom applications. Moving forward, translation pedagogy must move beyond motivation and actively cultivate students’ ability to regulate their own learning, refine their strategies, and adapt to new challenges. As self-regulation is the stronger predictor of translation competence, translation training programs should shift their focus from merely fostering motivation to actively developing self-regulation skills. While motivation can be nurtured through engaging and meaningful tasks (Dörnyei and Ushioda, 2021), students ultimately need structured guidance in goal-setting, self-monitoring, and strategy adjustment to sustain long-term translation competence. By doing so, educators can empower students not only to become skilled translators but also to develop lifelong learning competencies essential for the rapidly evolving translation industry.

### 6.3 Limitations and suggestions

While this study provides valuable insights into the role of self-regulation and motivation in translation learning, several limitations must be acknowledged.

First, the study was conducted within an undergraduate translation program in a flipped classroom setting, which may not fully capture the learning dynamics in other instructional contexts, such as professional translator training or postgraduate programs. Future research should explore whether similar patterns emerge across different educational levels and institutional settings.

Second, while this study controlled for prior translation experience, other external factors—such as instructor feedback, peer collaboration, and cultural attitudes toward self-regulated learning—may influence students’ ability to develop self-regulation. A longitudinal approach tracking students’ progress over time could provide deeper insights into how self-regulation evolves in translation learners. Finally, while this study employed multiple regression analysis to examine the direct effects of motivation and self-regulation on translation competence, it is acknowledged that this method captures only linear, direct relationships. Future research is encouraged to apply Structural Equation Modeling (SEM) to investigate more complex interrelationships among variables. SEM allows for the simultaneous analysis of both direct and indirect effects and can better account for latent constructs, such as metacognitive awareness or academic engagement, which may mediate or moderate the influence of self-regulation and motivation. This would offer a more comprehensive understanding of the pathways through which learner characteristics impact translation competence within flipped classroom settings.

## Data Availability

The original contributions presented in the study are included in the article/supplementary material, further inquiries can be directed to the corresponding author.
